# Older adults with cancer in the city of São Paulo: what factors determine the place of death?

**DOI:** 10.11606/S1518-8787.2018052016410

**Published:** 2018-07-17

**Authors:** Adna Kelly Ferreira Leite, Karina Braga Ribeiro

**Affiliations:** I Faculdade de Ciências Médicas da Santa Casa de São Paulo. Programa de Pós-Graduação em Saúde Coletiva. São Paulo, SP, Brasil; II Faculdade de Ciências Médicas da Santa Casa de São Paulo. Departamento de Saúde Coletiva. São Paulo, SP, Brasil

**Keywords:** Aged, Neoplasms, mortality, Hospital Mortality Terminal Care, Palliative Care, Home Nursing, Length of Stay, Idoso, Neoplasias, mortalidade, Mortalidade Hospitalar, Assistência Terminal, Cuidados Paliativos, Assistência Domiciliar, Tempo de Internação

## Abstract

**OBJECTIVE:**

Investigate factors associated with death at home among older adults who died of cancer in a large city.

**METHODS:**

This is a descriptive study, including all cancer deaths (ICD C00-C97) occurring between 2006 and 2012, among residents of the city of São Paulo, 60 years of age or older. The data source was the Mortality Information System, and the proportion of deaths was calculated according to place of occurrence, gender, age, race/skin color, education, marital status, cancer type, hospital bed availability, and year of death. The chi-squared test was used to examine the associations between the place of death and sociodemographic and clinical variables. Logistic regression was used to identify factors associated with home death. Crude and adjusted odds ratios and the corresponding 95% confidence intervals were estimated.

**RESULTS:**

Most of the deaths occurred in hospitals (88.2%). There was a significant association between the place of death and the following variables: gender, race/skin color, education, age, marital status, cancer type, hospital bed availability, and year of death. In the multivariate analysis, all variables, except the availability of hospital beds, remained as independent predictors of death at home.

**CONCLUSIONS:**

There was a predominance of hospital deaths, with an increase in frequency in the period. Female gender, higher education, married or widowed status, and black race were associated with a decreased risk of death at home, while increasing age, Asian race, and solid neoplasms were associated with higher risk of dying at home.

## RESUMO

**OBJETIVO:**

Investigar os fatores associados ao óbito domiciliar entre idosos que morreram por câncer em uma cidade de grande porte.

**MÉTODOS:**

Estudo descritivo, incluindo todos os óbitos por câncer (CID C00-C97) ocorridos entre 2006 e 2012, entre residentes do município de São Paulo com 60 anos de idade ou mais. A fonte de dados foi o Sistema de Informações de Mortalidade e a proporção de óbitos foi estimada segundo local, sexo, faixa etária, raça/cor, escolaridade, estado civil, tipo de câncer, disponibilidade de leitos hospitalares e ano do óbito. O teste qui-quadrado foi utilizado para investigar as associações entre o local do óbito e as variáveis sociodemográficas e clínicas. A regressão logística foi empregada para identificar fatores associados à morte domiciliar. Foram estimadas as razões de chance brutas e ajustadas e os intervalos de confiança de 95%.

**RESULTADOS:**

A maioria dos óbitos ocorreu em hospitais (88,2%). Houve associação significativa entre o local de óbito e as seguintes variáveis: sexo, raça/cor, escolaridade, faixa etária, estado civil, tipo de câncer, disponibilidade de leitos hospitalares e ano do óbito. Na análise multivariada, todas as variáveis, exceto a disponibilidade de leitos hospitalares, permaneceram como preditores independentes de óbito domiciliar.

**CONCLUSÕES:**

Houve predomínio de óbitos hospitalares, com aumento na frequência no período. O sexo feminino, maior escolaridade, o status de casado ou viúvo e a raça negra foram associados à menor chance de óbito domiciliar, enquanto o aumento da idade, a raça/cor amarela e as neoplasias sólidas estiveram associados à maior chance de morrer em casa.

## INTRODUCTION

Population aging is a global phenomenon, occurring not only in developed countries but also in developing countries. One of its consequences is the increase in the occurrence of chronic noncommunicable diseases, particularly cardiovascular diseases, cancer, and diabetes, which will account for more than 75% of all deaths worldwide^1^.

In Brazil, there was a rapid demographic transition, and in the city of São Paulo, between 1980 and 2012, the proportion of older adults in the population practically doubled, from 6.3% to 11.9%^2^.

In Brazil, for the year 2016, an estimated 600,000 new cases of cancer were estimated for both genders^3^_,_ and 196,954 deaths from the disease were recorded in 2013^4^. While about 59% of new cases registered in Brazil affect individuals over 60 years of age^5^, more than two-thirds of deaths are recorded in older adults^4^. The city of São Paulo has high of cancer incidence rates (293.2 and 208.3/100,000 inhabitants, adjusted rates for the world population, all neoplasms except skin)^5^ and neoplasms were the second cause of death among older adults residents in the city (20.7%), surpassed only by diseases of the circulatory system^4^.

Due to the increase in cancer incidence and mortality in this age group, many studies have addressed the issue of the place of death for all age groups^6–15^. However, the literature on this subject is still quite controversial concerning older adults. While some studies indicate a higher frequency of home deaths for this population group^8^ ^,^ ^9^, others indicate a higher frequency of hospital deaths^6^ ^,^ ^7^ ^,^ ^14^.

The city of São Paulo is one of the largest cities in the world, and the older population almost doubled in the period from 1980 to 2012^2^. With the technological advance, medicalization costs have been increasing, and many resources have been spent on patients at the end of their lives^16^. The identification of factors associated with place of death in the older population with cancer in the largest city in the country, as well as the monitoring of changes in these patterns, can help formulate public policies and allocate resources related to palliative care^11^.

## METHODS

This is a descriptive study using all cancer deaths (as the underlying cause, ICD-10 C00–C97) occurred in individuals aged ≥ 60 years old, living in the city of São Paulo, from 2006 to 2012. The data source is the Mortality Information System (MIS). Mortality data in the city of São Paulo are considered to be of good quality. During the study period (2006–2012) the percentage of unknown values for the variables gender (0.01%), age (0.3%) and race/skin color (4.1%) was small, as well as the percentage of deaths due to ill-defined causes (only 1.5% of all deaths)^4^.

The following data were retrieved from the MIS databases: place of death (hospital, home, other health facilities, public road, others, unknown); year of death; administrative district of residence; ICD-10 (hematological malignancies = ICD-10 codes (Leukemias and Lymphomas = C81 to C97) and solid tumors = ICD-10 codes (C00 to C79), age group, categorized as: 60-69 years, 70-79 years, 80-89 years, 90 years or more; gender (male, female); race/skin color (white, black, yellow/Asian, mixed, indigenous, unknown); education (years of study completed) (none, 1 to 3, 4 to 7, 8 to 11, 12 and over, unknown); marital status (single, married, widower, separated or divorced, unknown); and number of beds/1,000 inhabitants per district of residence (for the year 2012) (the cut-offs for this variable were established based on the quartiles)^17^.

Bivariate analyses were restricted to deaths in hospitals and households (n = 61,573, corresponding to 97.2% of all cancer deaths among older people in the period) and in all analyses the unknown values were excluded (for each variable).

The association between the independent variables and the place of death was evaluated in the bivariate analysis by the chi-squared test. Crude odds ratios and corresponding 95% confidence intervals were estimated. Multiple logistic regression was used to identify the independent factors associated with death at home and calculation of adjusted odds ratios. In the multiple model, we have included all variables that had a p < 0.25 in the bivariate analysis, but only those with p < 0.05 remained in the final model. The goodness of fit of the final model was assessed using the Hosmer and Lemeshow test.

We have calculated the Annual Percent Change (APC) in the proportion of home deaths in the period 2006–2012 using the Joinpoint method, having the calendar year as the regressor variable.

Statistical analyses were performed with the software Stata for Mac version 13.0 and Joinpoint Regression Program version 3.3. For all statistical tests, an alpha error = 5% was established.

## RESULTS

In the period of 2006–2012, 63,343 cancer deaths were recorded among older adults living in the city of São Paulo. The age of the individuals ranged from 60 to 104 years (median = 74 years) and there was a predominance of males (51.3%), white (75.7%), and married individuals (47.4%), with one to three complete years of education (25.1%) (Table 1). Most of the deaths occurred in hospitals (88.2%).

**Table 1:** Number and percentage of older adults who died from cancer according to sociodemographic variables. São Paulo City, 2006–2012.

Variable	n	%
Gender		
Male	32,532	51.3
Female	30,811	48.6
Race/Skin color		
White	47,989	75.7
Black	3,078	4.8
Asian	1,973	3.1
Mixed	7,053	11.1
Indigenous	20	0.0
Unknown	3,230	5.1
Age group (years)		
60–69	21,474	33.9
70–79	23,022	36.3
80–89	15,673	24.7
90 or more	3,174	5.0
Marital status		
Single	7,264	11.4
Married	30,044	47.4
Widowed	20,154	31.8
Separated/Divorced	3,965	6.2
Civil partnership	237	0.3
Unknown	1,679	2.6
Education (years of study)		
0	5,714	9.0
1–3	15,953	25.1
4–7	14,520	22.9
8–11	9,555	15.0
12 or more	7,791	12.3
Unknown	9,810	15.4
Year of death		
2006	8,471	13.4
2007	8,619	13.6
2008	8,813	13.9
2009	8,890	14.0
2010	9,266	14.6
2011	9,550	15.1
2012	9,734	15.4

Lung cancer accounted for 14% of cancer deaths among older adults. Among men, the highest frequencies were observed for deaths from lung (16.2%), prostate (15.1%), colon or rectum (10.9%), stomach (9.8%) and pancreatic cancer (5.0%). In females, there was a predominance of deaths due to breast cancer (15.2%), followed by malignant neoplasms of the colon or rectum (13.1%), and lung (10.9%) (Table 2).

**Table 2:** Number and percentage of older adults who died from cancer according to the type of malignant neoplasm (10 most frequent neoplasms) and gender. São Paulo City, 2006–2012.

Type of neoplasm (ICD-10)	Gender (%)	

	Male	Female
	
	n (%)	n (%)
Lung (C34)	5,280 (16.2)	3,365 (10.9)
Prostate (C61)	4,918 (15.1)	-
Colon/Rectum (C18-C20)	3,558 (10.9)	4,046 (13.1)
Stomach (C16)	3,182 (9.8)	2,010 (6.5)
Pancreas (C25)	1,625 (5.0)	2,100 (6.8)
Liver (C22)	1,527 (4.7)	1,225 (3.9)
Esophagus (C15)	1,386 (4.3)	420 (1.4)
Bladder (C67)	1,265 (3.9)	537 (1.7)
Mouth and oropharynx (C00-C06, C09-C10, C14)	1,204 (3.7)	360 (1.2)
Larynx (C32)	1,063 (3.3)	158 (0.5)
Breast (C50)	35 (0.1)	4,697 (15.2)
Ovary (C56)	-	1,244 (4.0)
Central nervous system (C70-C72)	810 (2.5)	963 (3.1)
Non-Hodgkin’s lymphoma (C82-C85)	791 (2.4)	949 (3.1)
Cervix uteri (C53)	-	827 (2.7)
Other neoplasms	5,888 (18.1)	7,910 (25.7)

Total	32,532 (100.0)	30,811 (100.0)

There was a significant association between gender and place of death (p < 0.001), with a lower frequency of home deaths among (8.8%) (OR = 0.89, 95%CI 0.85–0.95). There was also a significant association between the marital status and the place of death, with singles (10.0%) and widowers (10.0%) as the categories with higher frequencies of home deaths, compared to married or separated/divorced (p < 0.001). Education was also associated with the place of death, and older people with 12 years or more of education presented the higher percentage of home deaths (9.2%). We have also observed that, with aging, there was an increase in the number of older persons with cancer dying at home (16.1% for the group aged 90 years or older) (p < 0.001). There was also a significant association between race/skin color and the place of death, with Asians (13.8%) presenting a higher risk of dying at home (OR = 1.55, 95%CI 1.36–1.78, compared to whites), while those Blacks/mixed had a lower frequency of death at home (8.0%), compared to whites (9.8%) (OR = 0.84, 95%CI 0.78–0.91). In addition, there was a significant association between the type of neoplasm and the place of death, with a higher frequency of home death among those with solid tumors (9.5%) compared to those with hematological malignancies (4.8%) (OR = 2.09, 95%CI 1.83–2.41). Finally, we observed a statistically significant association between the availability of hospital beds in the district of residence and the place of death. The extreme categories, that is, with the lowest (zero bed/1,000 inhabitants) and the highest availability of beds (≥ 3.82 beds/1,000 inhabitants) were the ones that presented the highest percentages of home deaths (9.7% and 9.6%, respectively) (Table 3).

**Table 3:** Number and percentage of cancer deaths among older persons, according to the place of death (hospital *versus* home) and sociodemographic variables. São Paulo City, 2006–2012.

Variable	Place of death		OR (95%CI)	p

	Hospital	Home		
	
	n (%)	n (%)		
Gender				< 0.001
Male	28,600 (90.3)	3,070 (9.7)	1.00	
Female	27,274 (91.2)	2,629 (8.8)	0.89 (0.85–0.95)	
Marital status				< 0.001
Single	6,331 (90.0)	703 (10.0)	1.00	
Married	26,999 (91.3)	2,568 (8.7)	0.87 (0.80–0.94)	
Widowed	17,559 (90.0)	1,960 (10.0)	1.02 (0.93–1.11)	
Separated/Divorced	3,512 (91.8)	315 (8.2)	0.82 (0.71–0.94)	
Education (years of study)				< 0.001
None	5,144 (92.6)	413 (7.4)	1.00	
1–3	14,242 (92.3)	1,186 (7.7)	0.59 (0.53–0.64)	
4–7	12,849 (91.3)	1,212 (8.7)	0.67 (0.62–0.72)	
8–11	8,588 (92.1)	728 (7.9)	0.60 (0.55–0.66)	
12 or more	6,904 (90.8)	700 (9.2)	0.72 (0.66–0.79)	
Age group (years)				< 0.001
60–69	19,409 (92.7)	1,538 (7.3)	1.00	
70–79	20,495 (91.4)	1,923 (8.6)	1.18 (1.10–1.27)	
80–89	13,428 (88.5)	1,749 (11.5)	1.64 (1.53–1.77)	
90 or more	2,542 (83.9)	489 (16.1)	2.43 (2.17–2.71)	
Race/Skin color				< 0.001
White	42,071 (90.2)	4,556 (9.8)	1.00	
Black/Mixed	9,043 (92.0)	789 (8.0)	0.84 (0.78–0.91)	
Asian	1,650 (86.2)	264 (13.8)	1,55 (1.36–1.78)	
Indigenous	19 (100.0)	0 (0.0)	NC	
Year of death				< 0.001
2006	7,476 (89.7)	859 (10.3)	1.00	
2007	7,586 (89.4)	893 (10.5)	1.02 (0.93–1.13)	
2008	7,813 (90.1)	855 (9.9)	0.95 (0.86–1.05)	
2009	7,968 (10.0)	790 (9.0)	0.86 (0.78–0.95)	
2010	8,259 (91.6)	762 (8.4)	0.80 (0.72–0.89)	
2011	8,353 (91.3)	793 (8.7)	0.83 (0.75–0.91)	
2012	8,419 (91.9)	747 (8.1)	0.77 (0.69–0.86)	
Type of neoplasm				< 0.001
Hematologic	4,385 (95.2)	219 (4.8)	1.00	
Solid	48,797 (90.5)	5,115 (9.5)	2.09 (1.83–2.41)	
Number of hospital beds in the district of residence/1,000 inhabitants				0.004
0	13,826 (90.3)	1,490 (9.7)	1.01 (0.94–1.09)	
0.01–1.46	13,388 (91.3)	1,279 (8.7)	0.89 (0.83–0.97)	
1.47–3.81	15,285 (91.0)	1,504 (8.9)	0.92 (0.85–0.99)	
3.82–38.95	13,375 (90.4)	1,426 (9.6)	1.00	

NC: Not calculated

In the multivariate analysis, except for the availability of hospital beds, all other variables (gender, age, marital status, race/skin color, education, year of death and type of neoplasm) remained as predictors of death at home (Table 4).

**Table 4:** Crude and adjusted odds ratios (95% confidence interval) for factors associated with home death among older person with cancer. São Paulo City, 2006–2012.

Variable	Category	Crude OR	Adjusted OR	95%CI
Gender				
	Male	1.00	1.00	Ref.
	Female	0.89	0.82	0.77–0.88
Marital status				
	Single	1.00	1.00	Ref.
	Married	0.87	0.84	0.77–0.92
	Widowed	1.02	0.90	0.83–0.99
	Separated/Divorced	0.82	0.88	0.77–1.02
Education (years of study)				
	None	1.00	1.00	Ref.
	1–3	0.59	0.58	0.54–0.63
	4–7	0.67	0.67	0.62–0.73
	8–11	0.60	0.59	0.54–0.65
	≥ 12 years	0.72	0.72	0.65–0.79
Age group (years)				
	60–69	1.00	1.00	Ref.
	70–79	1.18	1.18	1.09–1.27
	80–89	1.64	1.63	1.51–1.76
	≥ 90	2.43	2.29	2.03–2.58
Race/Skin color				
	White	1.00	1.00	Ref.
	Black/Mixed	0.84	0.84	0.77–0.91
	Asian	1.55	1.51	1.32–1.74
	Indigenous	NC	NC	-
Type of neoplasm				
	Hematologic	1.00	1.00	Ref.
	Solid	2.09	2.11	1.83–2.42
Hospital beds/1,000 inhabitants				
	0	1.01	1.07	0.98–1.16
	0.01–1.46	0.89	0.93	0.86–1.02
	1.47–3.81	0.92	0.96	0.88–1.04
	3.82–38.95	1.00	1.00	Ref.

Ref.: reference; NC: not calculated

Hosmer and Lemeshow goodness-of-fit, χ^2^ = 2320.92, p = 0.520.

It was also observed that, in relation to the year of death, the deaths of older adults with cancer at home decreased significantly over time, from 10.3% in 2006 to 8.1% in 2012 (APC = -4.38%, p < 0.001) (Figure 1).

**Figure:**
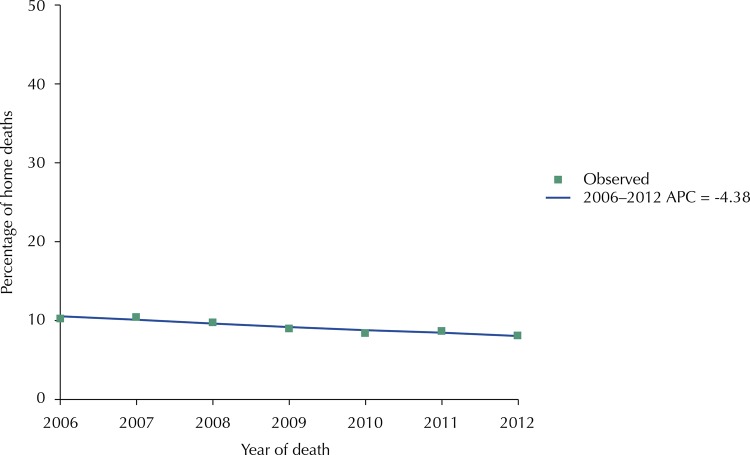
Time trends on the percentage of home deaths among older persons with cancer. São Paulo City, 2006–2012.

APC: Annual Percent Change

## DISCUSSION

This population-based study showed that, among older adults with cancer living in the city of São Paulo, most of the deaths occurred in hospitals and that only 9% of deaths occurred at home. This is the first study that analyzes the place of death among individuals with cancer, focusing on the older population residing in a large Brazilian metropolis.

Our results corroborate findings from other studies. A survey conducted in England, which included all cancer deaths recorded from 1993 to 2010, also reported a predominance of hospital deaths among adults over 24 years of age (48%)^14^. However, our results are not concordant with findings from studies conducted in Mexico City and Italy, where there was a predominance of home deaths (54% and 57.9%, respectively)^8^ ^,^ ^9^. Although except for the Mexican and Italian studies, a predominance of hospital deaths was observed, the magnitude of the difference between the proportion of hospital and home deaths in our study was significantly higher. However, it is worth noting that none of the previously mentioned studies included only the older population.

The results of our study showed that, in the city of São Paulo, older women who died from cancer had an 11% lower chance of dying at home compared to men. These findings are similar to those of a European study which reported that women living in the Netherlands, Norway, England, and Wales were less likely to die at home compared to men^10^. However, our findings are different from those observed in Canadian^7^ and UK^14^ studies, which recorded a higher percentage of home deaths among women. A possible explanation for the lower frequency of home death among women with cancer would be the lack of a husband or partner to care for them at home since the life expectancy is higher for women^15^. The presence of a gender issue is also considered since studies in the general population show a higher probability of death at home when the primary caregiver is a woman; besides, women are more often placed in the position of caregivers than in the position of recipients of care. However, it is essential to consider that it is not only a matter related to gender but is also necessary to consider the cultural and family context of these women^18^.

This study demonstrated that married or separated/divorced older adults with cancer had a lower chance of dying at home compared to single people. These findings are opposed to the results of a study developed in London, where it was observed that married individuals had a higher chance of dying at home than singles^19^. Cohen et al.^10^ also reported that married cancer patients living in Belgium, the Netherlands, and Norway had a higher chance of death at home and the same was observed for widowers residing in the Netherlands and Norway. However, it is important to note that, in that study, the authors used separated or divorced individuals as the reference category^10^. Apparently, marital status is a mediator of gender differences in the care for terminally ill patients. Differences in the place of death can be explained by the discrepancies in the availability of informal caregivers, and the literature has shown that women more often play this role^20^.

Our results showed a significant association between death and schooling, with a higher frequency of home deaths among older adults with higher education. Therefore, our data are similar to those described in Belgium, Italy, and Norway, where individuals with cancer and higher education had up to a 98% higher chance of dying at home, compared to individuals with a level of education less or equal to elementary school^10^. In the international study that evaluated the place of death for cancer patients residing in 14 countries and four continents, a higher level of schooling was associated with a higher chance of home death in the European countries (Italy, Spain, Belgium, and the Czech Republic), while the opposite was observed in Mexico, the United States, and South Korea^11^. We believe that the observation of the increase in the frequency of home deaths concomitant with the increase in education can be seen as an indicator of the possibility (or not) of payment for private home care.

In our study, there was an increase in home deaths concomitant with increasing age. These findings are similar to those described in a Mexican study, where older adults with cancer died more frequently in the households^9^. In contrast to these findings, a European study reported an increase in the frequency of home deaths, concomitantly with the increase in age only in Italy^10^, attributing this fact to the insufficient number of long-term care institutions for older population in that country, as this is still a responsibility of families^21^.

In Brazil, the legislation establishes that the care of dependent members should be a responsibility of the families. According to the National Sanitary Surveillance Agency (ANVISA), long-term care institutions for older people are public or private institutions, designed to serve as a collective residence for people aged 60 years and above, with or without family support, in a condition of freedom, dignity, and citizenship. Despite this official definition, there is still no consensus on what a long-term institution for older population is, and since its origin is linked to asylums (institutions initially created by Christian charity for the underprivileged population), there is some prejudice against this model of care^22^. The relationship between age and home deaths found in our study could be explained by this context.

Our study revealed that older persons with Asian ancestry had a higher frequency of home deaths compared to the other races (white, black/mixed and indigenous). Ethnic differences in the place of death among older people with cancer have been described in several studies. In the United States, according to the results of a study conducted in the Houston area, the risk of hospital death was higher among black individuals (OR = 1.51, 95%CI 1.37–1.66) compared to whites, Hispanics, and Asians^6^. In Japan, a national survey data published in 2000 revealed that approximately two-thirds of Japanese when diagnosed with a terminal illness, prefer to die in their homes. In addition, most Japanese identify themselves as Buddhists and Shintoists, whose dogmas claim that death is part of a natural process and that the person who dies will remain present in the family as a spirit^23^ ^,^ ^24^. In the last demographic census (2010), 2.2% of the inhabitants of the city of São Paulo declared themselves Asians, and among older adults, this percentage increases to 4.4%. The city of São Paulo has about one million Japanese and their descendants, and our results may be reflecting the cultural patterns of this population^25^.

Our findings corroborate the literature regarding the type of neoplasm. There is almost a consensus in the literature regarding the higher frequency of home deaths among those with solid tumors compared to those with hematological malignancies. It is known that, among individuals with hematological malignancies, hospital deaths are much more frequent because of the risk of intercurrences (bleeding, thromboembolic events, febrile neutropenia) and consequent need for antibiotics, more aggressive interventions and hospitalization after performing clinical procedures^6^.

Our results concerning the lack of association between the place of death and the availability of hospital deaths are not in agreement with the results of a Mexican study; in that study, a significant association between the density of public hospital beds and the place of death was recorded and also a higher chance of hospital death for those individuals living in places with higher bed availability^9^. On the other hand, in the study that analyzed the predictive factors of home death in Europe, there was no influence of the availability of hospital beds in this outcome in three countries (the Netherlands, Norway, and England)^10^. The authors pointed out that the relationship between the availability of hospital beds and place of death may not be so linear and possibly confounded by other factors, such as the availability of home care, cultural and religious issues, and even the communication between relatives^10^.

We observed that the frequency of home deaths among older adults with cancer decreased significantly over time, from 10.3% in 2006 to 8.1% in 2012. This result differs from most reports in the literature, which describes the reduction of hospital deaths over time^7^ ^,^ ^14^. Although it has been shown that the choice of cancer patients is to die at home^26^, preferences are not always respected and may reflect much more the practices and characteristics of the local health system. According to the World Health Organization, the provision of palliative care in Brazil is still isolated and disproportionate to the size of the population, and there is also a limitation in the availability of morphine^27^. It should be noted that the situation observed reflects the profile of the largest and most developed city in the country. In Brazil, in the same period, the percentage of home deaths for older adults with cancer ranged from 10.5% (Federal District) to 52.0% (Piauí)^4^. This large variation raises the hypothesis that the factors associated with home death in this group may be entirely different in distinct regions of the country.

The possible bias arising from unknown data in the death certificate and underreporting of cancer deaths can be listed as limitations of our study. However, mortality data in the city of São Paulo has reliable quality, and we do not believe that the results of the study would be substantially distorted.

Our results bring to light the need for a closer look at the quality of end-of-life care for people with cancer. We hope that the information generated in this study can be used to support public policies aimed at improving the offer of palliative care for older persons with cancer, benefiting both patients and their families with quality of life.
